# Advances in assessing Ca, K, and Mn translocation in oak tree stems (*Quercus* spp.)

**DOI:** 10.1016/j.heliyon.2024.e32627

**Published:** 2024-06-13

**Authors:** Caroline Christina Jaozandry, Jean-Michel Leban, Arnaud Legout, Gregory van der Heijden, Philippe Santenoise, Gilles Nourrisson, Laurent Saint-André

**Affiliations:** a INRAE, UR 1138 BEF, 54280, Champenoux, France; b Université de Lorraine, AgroParisTech, INRAE, UMR Silva, 54000, Nancy, France

**Keywords:** Nutrient translocation, Calculation chain, Tree rings, Drying method, ITRAX, ICP-OES

## Abstract

As a part of the biogeochemical cycle, nutrient translocation plays an important role in enhancing the capacity of perennial plants to grow in nutrient-poor soils. Although leaf translocation has been extensively studied, nutrient translocation between wood rings has received considerably less attention, primarily because of methodological constraints. This study aimed to (i) evaluate the effects of different drying techniques on Ca, K, and Mn concentrations, (ii) calibrate a semi-quantitative method for obtaining ring-to-ring nutrient concentrations along wood cores, and (iii) develop a complete calculation chain for nutrient translocation. Three pairs of cores per tree were extracted from nine oaks, and three drying methods—103 °C, 65 °C, and freeze-drying—were applied to each core pair. For each core pair, the first core was analyzed using ITRAX. The second core was analyzed using ICP-OES following the mineralization of a 20 mg wood sample. Ca, K, and Mn concentrations and wood density were not affected by the drying methods (*p* > 0.05 for Ca, K, and Mn). After upscaling at the stand level, the total translocation was 10.8 ± 5.5 kg ha^−1^, 14.8 ± 11.4 kg ha^−1^, and 2.6 ± 0.9 kg ha^−1^ for Ca, K, and Mn, respectively, after 45 growing years. The total Ca, K, and Mn translocation showed a strong tree effect, partly explained by tree diameter. The study findings suggest that similar measurements can be performed on all wood cores sampled in previous studies and stored after air-drying. These results provide a reference for future analyses of Ca, K, and Mn translocations in different species from wide geographic areas.

## 1 Introduction

Forest capacity for atmospheric carbon sequestration allows them to contribute efficiently to climate change mitigation [1,2]. However, forest carbon sinks are heavily affected by drought events, high temperatures [3], and intensive forest biomass harvesting [4]. The demand for forest products is increasing, particularly for energy purposes [5] impeding their sustainability. Forest capacity to provide these services is strongly linked to biogeochemical cycles [6].

The concept of biogeochemical cycles encompasses geochemical, biological, and biochemical components [7]. This last cycle is fundamental to perennial plants, especially trees, because it allows nutrient recycling within the plant. This reduces soil nutrient uptake [8]. Translocation is defined as the remobilization of nutrients from senescent to newly growing plant tissues [9]. It is primarily based on three processes, that is, use of nutrients stored in vacuoles, degradation of storage proteins, and breakdown of cellular structures by enzymes, thereby removing structurally bound nutrients [10]. Mineral element translocation allows trees to maintain their growth in low-fertility soils [11,12] by reducing their dependence on soil nutrients and optimizing available nutrient consumption during biogeochemical cycling [13].

Foliar translocation has been widely documented [[14], [15], [16]]. However, there have been relatively few studies focusing on nutrient translocation in other tree organs, and especially wood. Knowledge of translocation dynamics in tree stems is crucial for achieving a balance in biogeochemical cycling [13]. In tree stems, translocation is the radial transfer of nutrients from the old to newly formed rings. The transfer of mobile elements from heartwood to sapwood explains the lower nutrient concentrations observed in heartwood than in sapwood [17,18]. One method for measuring nutrient translocation in wood stems is isotope tracing. This method provides information on the percentage of nutrients transferred from old to new tissues via isotope injection into the plants [[19], [20], [21], [22]]. Nevertheless, this technique does not provide a ring-by-ring translocation value during tree life, owing to isotope dilution. Another method is to sample tree rings over part of the tree's lifespan or its entire lifespan [8,13,23]. However, this method requires sampling over the entire stand rotation. This may be feasible in fast-growing stands, that is, six years for Eucalyptus plantations, but almost impossible for slow-growing species such as oak, with a revolution of more than 100 years. Modeling the evolution of nutrient concentration in one ring allows calculating the decrease in nutrient concentration in tree rings [24]. However, this remains a fraction of the complete translocation calculation. Therefore, methods for calculating nutrient translocation are either incomplete or difficult to apply.

To draw up a complete calculation chain for nutrient translocation in wood core samples, two methodological issues must be solved, namely, the drying method and ring-to-ring concentration measurements.

The quantification of nutrients in wood requires measuring the volume, wood density, and mineral element concentrations in each tree ring. The wood density and mineral element concentrations require adequate measurement methods, and the drying method is critical. Reference methods for drying vary between disciplines such as wood science, soil science, and ecology. The samples may be either air-dried e.g. Refs. [[25], [26], [27]], oven-dried at 65 °C e.g. Refs. [28,29], or dried at 103 °C [30] or freeze-dried [31]. In multidisciplinary studies that combine wood density and nutrient concentration, this is a major issue because drying wood samples at 103 °C, such as for reference in wood sciences, and especially for measuring wood density, may bias the measurement of nutrients in wood owing to nutrient volatility and transfer of dissolved elements with water mass flow. To the best of our knowledge, the influence of different drying methods on the measured nutrient concentrations in wood has not yet been reported.

A diachronic approach allows the measurement of wood translocation. However, sampling incremental cores each year in the same tree is not feasible. This is because repeated coring of the same tree rapidly becomes lethal. Therefore, the estimation of translocation is conducted in stands of different ages with similar climatic and ecological conditions [8,13]. This is performed by felling trees to collect stem wood disks or sample wood cores. In these samples, the measurement of nutrient content from the pith to the bark involved both nondestructive and destructive methods.

The X-ray fluorescence microanalysis spectrometer (μ-XRF) is the most widely used nondestructive method allowing the measurement of the relative variations in nutrient content along tree cores [25,[32], [33], [34]]. This method is directly applicable to the planned samples and requires minimal preparation time. In contrast, destructive methods such as inductively coupled plasma-optical emission spectrometry (ICP-OES) and inductively coupled plasma-mass spectrometry (ICP-MS) are time-consuming and require sample cutting, grinding, and mineralization [[35], [36], [37]]. They can be used to obtain results used for calibration by (i) analyzing a sample processed using XRF with ICP-OES [31] or (ii) grinding a sample. Part of this is analyzed by ICP-OES and the remainder is compressed into a pellet and passed through XRF [38]. Because a minimum of 200 mg per sample is required for the mineralization process preceding the ICP-OES analysis, the radial resolution is low and up to 10 annual rings/sample, thus masking annual variability. To the best of our knowledge, no study has yet tested the measurement of nutrient ring-to-ring concentrations in 20 mg samples.

This study focused on oak trees (*Quercus* spp.) because they are dominant in French hardwood forests. They represent 44 % of the hardwood standing volume [39]. The nutrients considered were calcium (Ca), potassium (K), and manganese (Mn).

Calcium is important for cell wall and membrane stabilization, osmoregulation, and as a second messenger that allows plants to regulate developmental processes in response to environmental stimuli [10]. Potassium is highly mobile and plays an important role in metabolic activity. Its main function is osmoregulation, which is important for cell extension and stomatal movement. Potassium further affects sucrose loading and the rate of solute movement within the plant based on mass flow [10]. Manganese is an important micronutrient for redox systems, as an activator of various enzymes, and for lignin synthesis [10].

Therefore, the first objective of this study was to determine whether different drying techniques affected the nutrient concentrations and wood density of the cores. The second objective was to measure the nutrient concentration at the ring level along the tree cores by calibrating the x-ray fluorescence (ITRAX) method with ICP-OES measurements. The third objective was to propose a step by step guide for calculating the within-tree stem translocation of nutrients over tree life.

## 2 Materials and methods

### 2.1 Experimental site

The work was conducted at the Champenoux experimental site located in North East France (latitude 48°43′17.5285″, longitude 6°20′26.8966″) at an altitude of 280 m. This site is included in the Manipulation of Soil Organic Matter (MOS) network of the national research infrastructure of IN-SYLVA, France [40]. This site was designed to study the effects of increased biomass export on soil fertility and biogeochemical functioning of the ecosystem [41]. The stand was primarily composed of oak (*Quercus petraea* L*.* and *Quercus robur* L.) mixed with hornbeam (*Carpinus betulus*). The trees were grown from natural regeneration, and were on average 45 years old [42], with a mean diameter at breast height (DBH) of 16.6 cm. The soil substrate consists of Pliensbachian marl and the soil is a Dystric Cambisol (WRB, 2014) with eumull/mesomull as humus. The experimental design was composed of three blocks of four 40 m* 40 m plots. This study focused on the oak trees in the control plot of Block 1 with 92 oak trees representing 575 stems ha^−1^.

### 2.2 Wood core sampling

A Pressler's increment borer (diameter = 5 mm) was used to sample 54 cores from nine trees at DBH (for more details, see Supplementary Material). Six diametric wood cores per tree were evenly distributed around the tree circumference to avoid bias owing to potential anisotropic growth. The six cores were considered repetitions for a single tree. After collection, the cores were placed in sealed polycarbonate boxes and stored at 5 °C to prevent fungal growth and drying. For each tree, the first pair of cores was oven-dried at 103 °C, the second at 65 °C until a constant weight was reached, and the third was freeze-dried for 48 h. For each pair, the first core was analyzed using the nondestructive method ITRAX. The second core was analyzed using ICP-OES, which is a destructive method.

### 2.3 Tree ring density measurement and mineral analysis using ITRAX

Density measurements were performed using an ITRAX multiscanner (Cox Analytical Systems). The 27 cores selected for nondestructive analysis were sawn into radial strips of 2 mm thickness using two parallel circular saws composed of tungsten carbide. The cores were stabilized at 12 % moisture after each drying method and scanned for obtaining one radiographic image per sample. Each x-ray image was processed using CERD software to detect the annual ring limits and to calculate the within ring wood density variations with a resolution of 24 μm [43]. The wood core was exposed to x-ray radiation in the line scan mode, and fluorescent photon count rates (relative concentration) were measured for Ca, K, and Mn along the entire core length. The measurement of wood mineral elements was performed along the same sample with a radial resolution of 200 μm.

### 2.4 Cross-dating the tree cores sampled

At the tree level, even close to each other, the six cores showed differences between them, resulting from small variations caused by borer orientation. Therefore, it was necessary to perform tree-by-tree and between-tree cross-dating to compare ring width and wood density patterns between cores. This was performed by counting and synchronizing the number of rings from the bark to the pith by determining the pointer years [44].

### 2.5 Sample selection and mineral analysis by ICP-OES following wet mineralization

Usually, the nutrient analysis of wood samples is based on a minimum of 200 mg of ground wood [45], which corresponds to 5–10 rings in oak trees [30]. With the objective of fitting the ITRAX resolution and calculating the annual ring translocation, the method was adapted to a lower amount of wood (10 times lower: 20 mg). This corresponds to a 1 mm wood ring width.

**Fig. 1:**
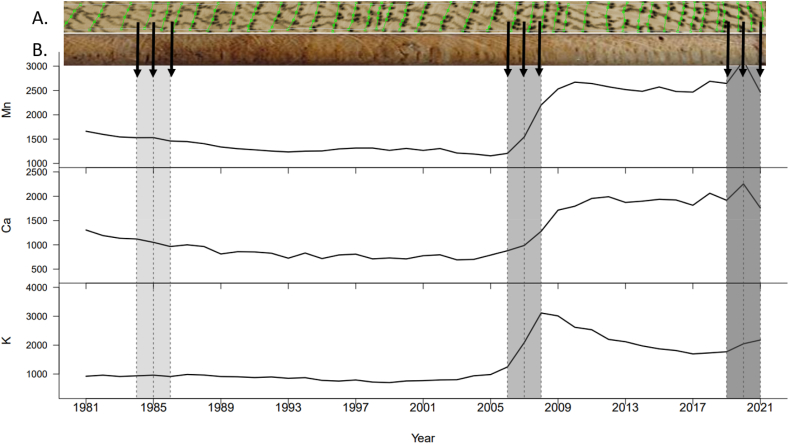
(A) Core selected for ITRAX analysis and (B) core selected for ICP-OES analysis. All cores have been cross-dated to ensure exact correspondence between ITRAX and ICP EOS analysis. The y axis indicates the X-ray intensity values, in ITRAX counts, that is, average counts of Ca, K, and Mn of each ring. The shaded area corresponds to the group of rings selected on (A) to be cut out on (B) for the destructive analysis. The light shade corresponds to the heartwood, the middle shade corresponds to the transition zone, and the dark shade corresponds to the sapwood. The core is oriented from heartwood to sapwood.

To calibrate the fluorescent photon count rates measured by the ITRAX, three 1 mm long sections were defined along each wood core selected for the ICP-OES analysis. These three sections represent the heartwood, transition zone, and sapwood (Fig. 1a and b). The final number of wood rings per section varied from one to three.

Each section was then separated from the wood core using a ceramic blade and was inserted into a borosilicate 16 mL glass, placed in Teflon vessels, and was digested with 69 % HNO_3_ in a closed microwave oven (Anton Paar Multiwave 5000, rotor 24HVT50). Afterward, the digested samples were diluted with Millipore water to obtain a 10 ml solution, which was analyzed using ICP-OES (Agilent 7000 series).

Given that the cores were taken from suppressed trees, the ring widths in the sapwood were relatively small. Therefore, many sapwood samples consisted of a group of two or three rings. Nevertheless, the rings in the heartwood and the transition zone were often wide, and one sample corresponded to one ring. The method developed in the present study enables the destructive analyses of wood chips at a close annual ring scale.

### 2.6 Step by step calculation of translocation (Equations (Equation 1), (Equation 2), (Equation 3), (Equation 4), (Equation 5), (Equation 6), (Equation 7), (Equation 8), (Equation 9), (Equation 10), (Equation 11)))

#### 2.6.1 Ring biomass

##### 2.6.1.1 Ring weighted wood density

Individual wood ring density was measured using an ITRAX across the tree radius of each ITRAX and ICP-OES wood core. Each diametric core was cut into two radii, and the density of each individual wood ring was measured using ITRAX. To consider the anisotropic growth of the trees, the average wood ring density was calculated by dividing the mean wood ring density by the ring width of each radius.

(Equation 1) $$d_{i}=\frac{d_{i,1}{RW}_{i,1}+d_{i,2}{RW}_{i,2}}{{RW}_{i,1}+{RW}_{i,2}}$$

$d_{i}\text{:}$ Density of ring i

$d_{i,1}$: Density of ring i in the first radius of the core

$d_{i,2}$: Density of ring i with the second radius of the core

${RW}_{i,1}$: Width of ring i in the first radius of the core

${RW}_{i,2}$: Width of ring i with the second radius of the core

##### 2.6.1.2 Stem volume

The model established by Deleuze et al. [46] was parameterized for any type of species and stage of tree development in France and was suitable for the present study.

(Equation 2) $$V_{t}=\frac{h\times {CBH}^{2}}{4\times \pi }\times \frac{\left(a+b\times \frac{\sqrt{CBH}}{h}+c\times \frac{h\;}{CBH}\right)}{\;{\left(1-\frac{1,30}{h}\right)}^{2}}\times \left(d+e.\log\left(\frac{1}{h}\right)+f\frac{\sqrt{CBH}}{h}+\frac{g}{CBH}\right)$$

$V_{t}\text{:}$ Volume of a tree at time t

h: Total tree height

hf: Height of first drop of decreasing diameter (first fork)

CBH: Circumference at breast height

##### 2.6.1.3 Ring volume

The volume of a ring is obtained by the difference between the volume of the tree at time *t* and time *t−1*:

(Equation 3) $$V_{i,t}=V_{t}-V_{t-1}$$

$V_{i,t}$: Volume of a ring i at time t

$V_{t}$: Volume of the tree at time t

$V_{t-1}$: Volume of the tree at time t−1 (previous year)

The biomass of a single ring i at time t is:

(Equation 4) $$B_{i,t}=V_{i,t}\times d_{i}$$

$V_{i,t}$: Volume of ring i at time t (m^3^)

$d_{i}$: Mean density of ring i (kg m^−3^) obtained from microdensitometric data

$B_{i,t}$: Biomass of ring i at time t (kg)

#### 2.6.2 Nutrient concentration in a ring

The nutrient concentration of a ring over time is calculated by:

(Equation 5) $$C_{{i,t,t}_{in}=I_{c}\;\exp^{{-k(t-t}_{in)}}+F_{c}\frac{\left({t-t}_{in}\right)}{t_{fin}}}$$

$t_{in}$: Age of the tree when ring i is formed (years)

$t_{fin}$: Age of the tree at the end of stand revolution (i.e. harvest) (year)

${t-t}_{in}$: Current age of the ring (year)

$F_{c}$: Final concentration of ring i measured using destructive methods (mean concentration of the rings close to the pith)

The first component of the model corresponds to the decrease in nutrient concentration over time, and the second component was introduced to separate $I_{c}$ from $F_{c}$. The main aim of this approach was to reduce the potential correlation between $I_{c}$ and $F_{c}$, and to take the rotation length ($t_{fin}$) explicitly into account in Equation (5) [24].

The initial concentration ($I_{c}$) of nutrients in a ring at *t = 0* is given by:

(Equation 6) $$I_{c}=a+\exp^{-bt_{in}}$$

**Fig. A1:**
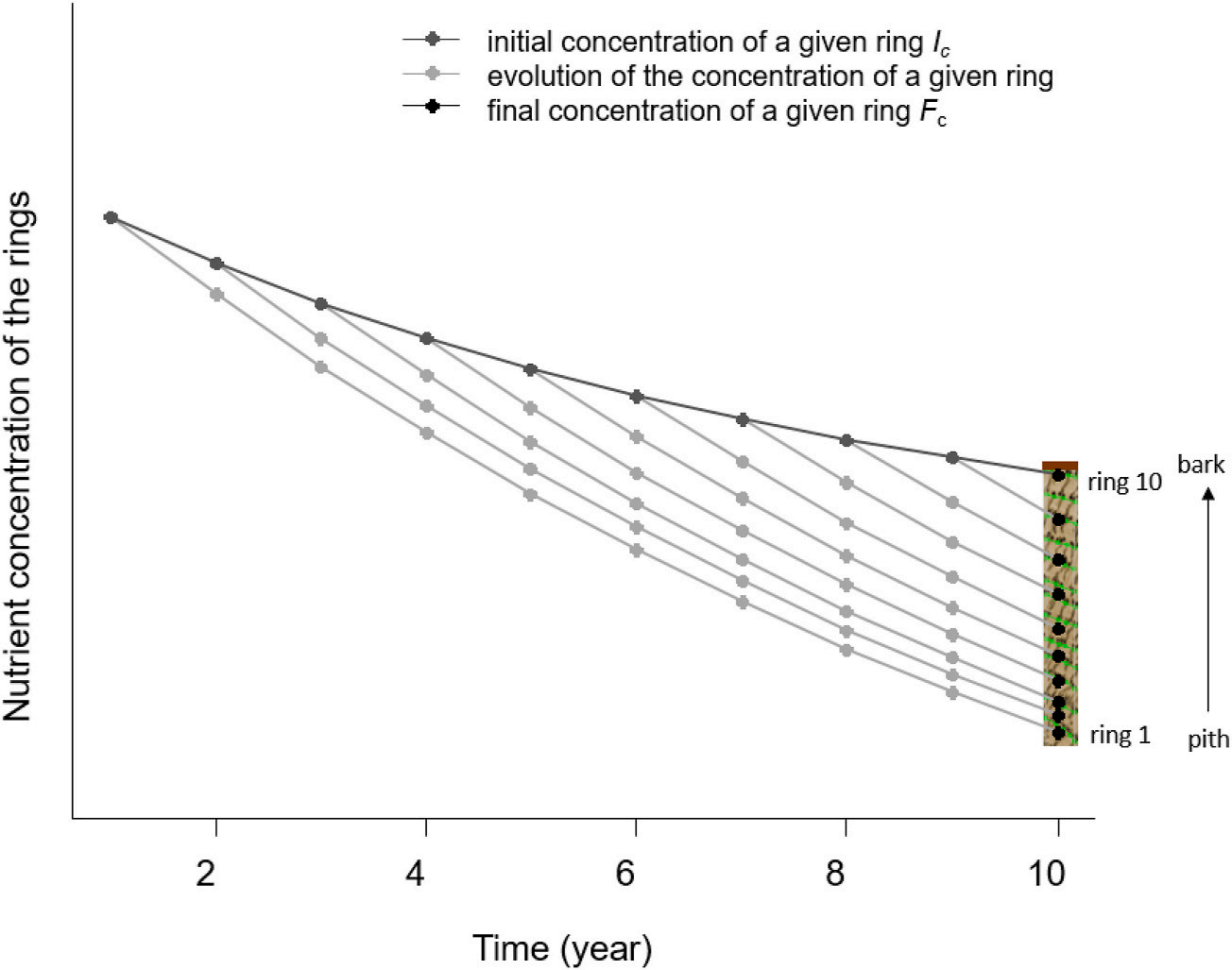
Example of the evolution of ring nutrient concentration for a 10 years old tree. Black points indicate the initial concentration of a ring at the time of ring initiation. Gray points indicate the evolution of the concentration of a ring over time.

$I_{c}$ was estimated as the nutrient concentration, which was assumed to decrease with age at the initiation of the tree ring [24] (see Fig. A1 in the Appendix).

a: Final concentration of the last ring in the core

Parameters k and b are nutrient-dependent and were adapted from Saint-André et al. [24], for oak trees, potassium: k *= 0.073;* b *= 0.1357*; calcium*:* k *= 0.2;* b *= 0.15* and manganese: k *=0.07;* b *= 0.15.*

#### 2.6.3 Nutrient pool in a ring

The nutrient pool in a single ring i at time t is:

(Equation 7) $$N_{i,t}=C_{i,t,t_{in}}B_{i,t}$$

$N_{i,t}\text{:}$ Nutrient pool in a ring i at time t (g)

$C_{i,t,t_{in}}$: Nutrient concentration in a ring i at time t (g kg^−1^)

$B_{i}$: Biomass of ring i at time *t* (kg)

#### 2.6.4 Nutrient translocation

The nutrient translocation in a ring year by year is calculated by:

(Equation 8) $$T_{i,t}=N_{i,t}-N_{i,t+1}$$

The total nutrient translocation in a ring is calculated by:

(Equation 9) $$T_{i}=\sum_{t=t_{in}}^{t_{fin}}\left(N_{i,t}-N_{i,t+1}\right)$$

The cumulative nutrient translocation in a tree with *n* rings over time was calculated as follows:

(Equation 10) $$T_{tree}=\sum_{i=1}^{n}T_{i}$$

The average cumulative translocation of the nine measured trees multiplied by the number of stems per hectare provided an approximate estimate of the nutrient cumulative translocation per hectare.

The relative cambial age was equal to zero at the pith and one for the last ring close to the bark. This enabled us to compare trees of different ages. The relative cambial age $A_{ri}$ of ring r of tree i was calculated as follows:

(Equation 11) $$A_{ri}=\frac{R_{n}}{A_{i}}$$

With $R_{n}=$ ring number n, $A_{i}=$ age of tree when the core was sampled.

### 2.7 Statistical analysis

#### 2.7.1 Tree ring mineral analysis by ICP-OES

For the destructive analysis, variations in the measurements made on 81 rings from three radial positions of the 27 cores were investigated (Fig. 1a and b). We aimed to identify the effect of the drying method on the concentrations of Ca, K, and Mn, after accounting for the effects of the ring radial position on each core. The ring position was used instead of cambial age because three groups of rings per core were analyzed.

The *lme4* package and *lmerTest* [47] from R studio software (R Core Team, 2021) were used to fit the linear mixed-effect model to the data. Ring position and drying method were considered as factors with oven-dried at 103 °C being the reference because it is the most used drying method for wood density measurements. A generalized linear model was used to establish a model with fixed effects of ring position and drying method. Several models were tested by progressively adding and interacting with the effects of the models. The model with the lowest AIC (Akaike Information Criterion) was retained. The random effect (the "tree" variable) was then added to the previously selected model. A model with fixed effects was selected, because the addition of random effects did not improve the model AIC. In order to determine whether the drying methods significantly affect the concentration of each mineral element, an analysis of variance (ANOVA) was performed. The mean nutrient concentrations of the ring positions were compared using post hoc multiple mean comparisons of Tukey's test in the *emmeans* package [48]. Letter displays were provided using the *multcomp* package [49]. The model is defined as follows.

(Equation 12) $$Y_{rijk}=\mu +{DM}_{j}+P_{k}+\varepsilon_{rij}$$

where $Y_{rijk}$ is the measurement (Ca, K, and Mn concentrations) of a ring *r* from tree *i* (*i = 1 to 9*) and dried at *j* (qualitative variable j *= 1: core oven-dried at 65 °C, 2: core oven-dried at 103 °C and 3: core freeze-dried; 1 and 3 are compared to 2, which is the reference drying method*) at radial position k (qualitative variable k *= A: heartwood, B: transition zone and C: sapwood; B and C are compared to A, which is the reference radial position*) (Fig. 1a and b)*,* μ is the overall mean*,* ${DM}_{j}$ is the drying method *j,* $P_{k}$*:* is the radial position *k* and $\varepsilon_{rij}$ is the residual random variation.

#### 2.7.2 Tree ring mineral analysis using ITRAX

Statistical analyses were performed on the 27 ITRAX wood cores with 742 rings. The purpose was to identify the effect of the drying method on wood density and concentrations of mineral elements (Ca, K, and Mn). Before starting statistical analysis, the relationship between each dependent and explanatory variable was checked for linearity. The log-transformed ring width was used to handle the nonlinear effects. Because the radial positions of sapwood and heartwood varied with tree age from 40 to 50 years, cambial age was normalized to tree age to obtain a relative age for each wood ring.

The model was built according to the process described in Section 2.7.1. To select the optimal model, a likelihood-ratio test was performed to compare the full model including the drying method with the restricted model. The number of parameters of some models could then be reduced by maintaining the restricted models when there was no difference between them (complete and restricted). Linear mixed models were constructed using the *lme4* package and *lmerTest* [47]. Decomposition of the total variance of the models from the partial $R^{2}$ ($R^{2}m$ and $R^{2}c$) was performed using the *MuMin* package [50]. The complete model is defined as follows:

(Equation 13) $$Y_{rij}=\mu +A_{rij}+{\ln\left(RW\right)}_{rij}+{DM}_{j}+\left(A_{rij}:{\ln\left(RW\right)}_{rij}\right)+\left(A_{rij}:{DM}_{j}\right)+\left(1+{A_{rij}+DM}_{j}+{\ln\left(RW\right)}_{rij}|T_{i}\right)+\varepsilon_{rij}$$

Where $Y_{rij}$ is the measurement (mean ring density, Ca, K and Mn relative concentrations) of the ring *r* of the tree *i* (*i = 1 to 9*) dried at *j* (qualitative variable j*= 1: core oven-dried at 65 °C, 2: core oven-dried at 103 °C and 3: core freeze-dried; 1 and 3 are compared to 2, which is the reference drying method*), μ is the overall mean, $A_{rij}$ is the relative cambial age of a ring *r* from tree *i* dried at *j*, ${\ln\left(RW\right)}_{rij}$ is the log-transformed ring width of the ring *r* from tree *i* and dried at *j*, ${DM}_{j}$ is the drying method *j,* $\left(A_{rij}:{\ln\left(RW\right)}_{rij}\right)$ is the interaction between relative cambial age and the ring width of a ring *r* from tree *i* dried at *j,* $\left(A_{rij}:{DM}_{j}\right)$ is the interaction between relative cambial age of a ring *r* from tree *i* dried at *j* and the drying method *j,* $\left(1+{A_{rij}+DM}_{j}+{\ln\left(RW\right)}_{rij}|T_{i}\right)$ is the random effect of the tree factor linked to the relative cambial age, the drying method and the ring width, $\varepsilon_{rij}$ is the residual random variation.

### 2.8 Calibration of the ITRAX method with the ICP-OES destructive measurements

**Fig. 2:**
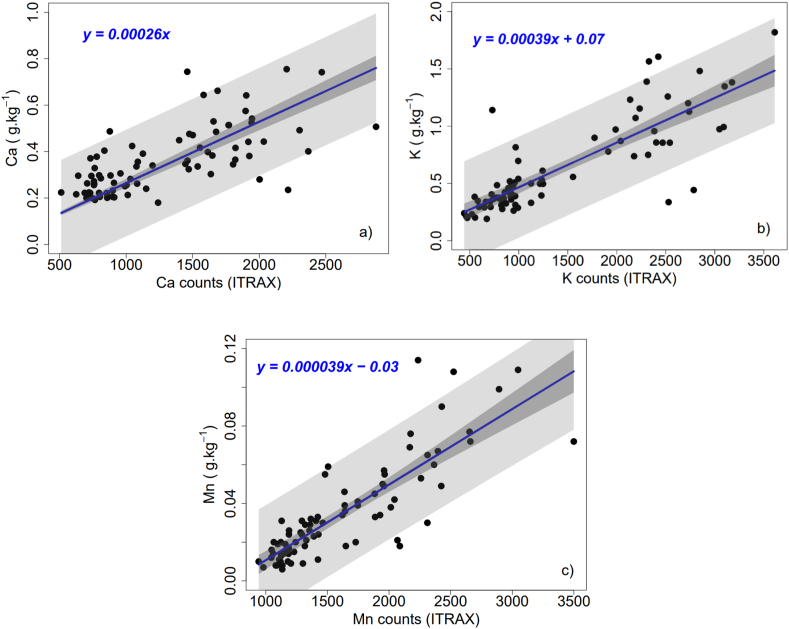
Linear regression model of conversion of semi-quantitative values into quantitative values for Ca (a), K (b), and Mn (c). The blue lines indicate the linear regression, the dark bands are the confidence interval for the mean, and the light bands indicate the confidence interval for the individual prediction.

From the cross-dated rings of the two cores in the ITRAX and ICP-OES analyses, linear regression models were tested to select the best-performing model for converting semi-quantitative data (ITRAX) into quantitative values of Ca, K, and Mn (Fig. 2a, b, c). This allowed the calculation of nutrient ring-to-ring translocation. For each nutrient studied, a global model grouping data from all the drying methods and one model per drying method were fitted. Fisher's test was applied to determine the significance of the differences between the global calibration model and each drying method model [51].

## 3 Results

### 3.1 Tree ring mineral analysis by ICP-OES

**Fig. 3:**
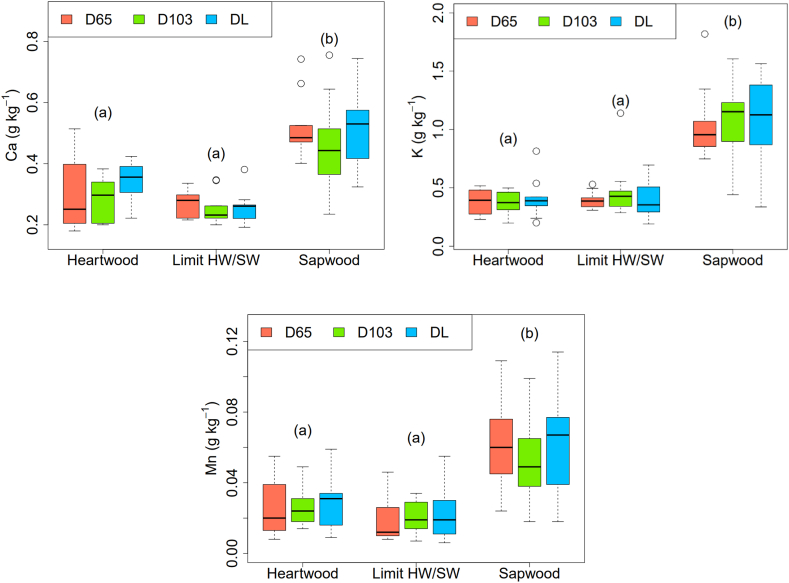
Ca, K and Mn concentrations for the three drying methods (D65 = drying at 65 °C, D103 = drying at 103 °C and DL = lyophilization or freeze-dried) according to the radial position in the tree: heartwood, limit HW/SW (transition zone) and sapwood. Different letters indicate a significant difference (p < 0.05; Tukey's Test).

With drying at 103 °C as a reference, the drying method had no significant effect on Ca, K, and Mn concentrations (*p* > 0.05). For the heartwood, transition zone, and sapwood, the nutrient concentration for Ca was 0.31 g kg^−1^, 0.26 g kg^−1^, 0.49 g kg^−1^, for K was 0.38 g kg^−1^, 0.42 g kg^−1^, and 1.07 g kg^−1^, and for Mn it was 0.026 g kg^−1^, 0.02 g kg^−1^, and 0.058 g kg^−1^, respectively. For the three mineral elements, concentrations were not significantly different in the heartwood and transition zones (*p* > 0.05, 0.06 for Ca; 0.5 for K and 0.2 for Mn). Meanwhile, concentrations in sapwood were significantly higher (*p* < 0.001, ***, 2.5 × 10^−9^ for Ca; 2.4 × 10^−16^ for K and 7.1 × 10^−8^ for Mn) (Fig. 3).

### 3.2 Measurement of tree ring wood density and nutrients by ITRAX

**Fig. 4:**
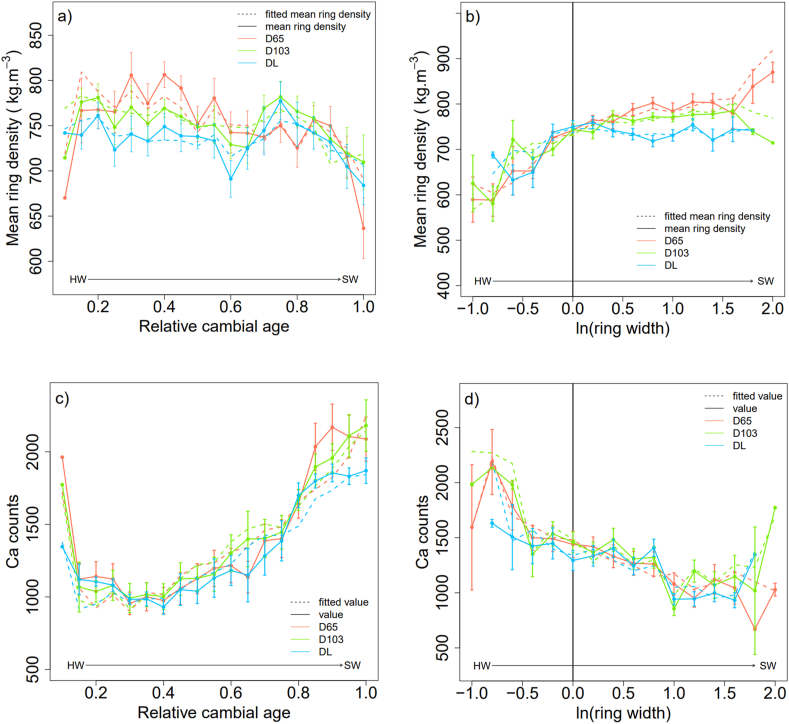

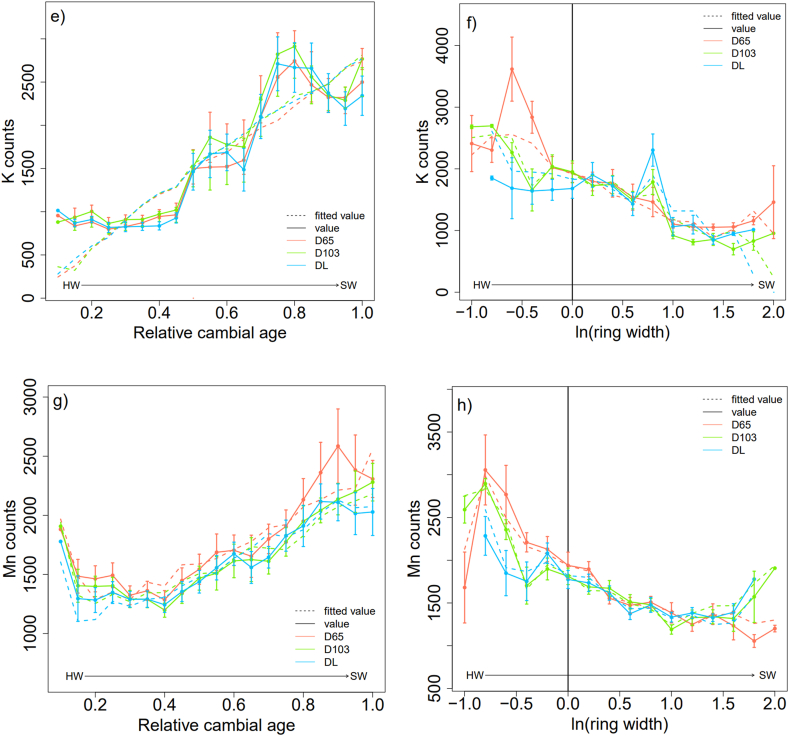
Ring mean density (kg.m^−3^), Ca, K and Mn counts according to relative cambial age (a, c, e, g) or ln (ring width) (b, d, f, h), ln (ring width) = −1 corresponds to ring width = 0.33 mm; ln (ring width) = 1 corresponds to ring width = 2.71 mm.

For the ring mean density, the effect of the drying method was not significant. Meanwhile, there was a significant and negative effect of the relative cambial age (*p* < 0.01, **), a positive effect of the logarithm of the ring width (*p* < 0.05, *), and a positive effect of the interaction between relative cambial age and the logarithm of ring width (*p* < 0.001, ***) (Table 1, Fig. 4a). The closer the ring was to the bark, the lower the density. A thicker ring has a higher density (Fig. 4b), which is modulated by the relative cambial age. The random effects were significant only for the intercept (Table 1). Taking into account the three effects (Table 1, Fig. 4a and **b**), the total explained variance was 79.51 %, of which 31.12 % was from the fixed effects and 48.39 % from the random effects (see Table A. 1 in Appendix).

**Table 1:** Linear mixed-effect model results on the tree rings wood density and mineral elements (Ca, K, Mn) according to the drying methods (D65, DL) with D103 as baseline, relative cambial age ($A_{r})$ and ring width logarithm ln(RW). Here are displayed the intercept and fixed effects estimates ± standard errors and statistical significance (with ***p < 0.001, **p < 0.01, *p < 0.05, . *p* < 0.1, non-significant (ns) *p* > 0.1) of the average model, number of observations = 742.

	Mean ring density		df	Ca		df	K		df	Mn		df
*intercept*	739.1 ± 20	***	10	287.3 ± 194.1	ns	9.4	280.44 ± 148.3	ns	27.1	579.3 ± 107.1	***	7.8
$A_{r}$	−31.3 ± 10.7	**	681.7	1917.8 ± 267.3	***	10.2	2677.6 ± 155.3	***	737.8	1748.7 ± 209.5	***	7.3
ln(RW)	33.5 ± 1	*	18.1	428.5 ± 65.7	***	656	−241.9 ± 118.6	ns	735.4	493.4 ± 75	***	31.6
$A_{r}$ × ln(RW)	116 ± 15.3	***	355.8	−837.5 ± 115.8	***	659	237.8 ± 203.7	ns	736.6	−947.5 ± 112	***	358.9
$A_{r}$ × DL				−421.9 ± 127.2	***	706.4

Calcium (Ca) concentration increased significantly with relative cambial age. Rings near the bark had a higher Ca concentration than those near the pith (*p* < 0.001; *** Fig. 4c). There was a highly significant and negative effect of the ring width logarithm on Ca (*p* < 0.001, ***, Fig. 4d) and wider rings had lower Ca concentrations. The effect of the drying method on Ca concentration was not significant. The interaction between the relative cambial age and ring width logarithm was significant and negative (*p* < 0.001, *** Table 1 and Fig. 4d). The model also indicated an interaction effect between relative cambial age and the freeze-drying method (*p* < 0.001). The random effect was significant for inter-tree variation of relative cambial age ($\delta_{T_{\text{Ar}}}^{2}\text{:}$ 19.63 %) and the intercept was slightly significant ($\delta_{T}^{2}\text{:}$ 11.16 %). The total variance explained was 73.16 %, with 37.24 % for fixed effects and 35.92 % for random effects (see Table A1 in Appendix, Fig. 4c and d).

Relative cambial age had a significant positive effect on the K concentration (*p* < 0.001, ***, Table 1 and Fig. 4e). The Ca and K concentrations increased when the ring moved closer to the bark. However, the effects of the other model parameters, that is, the drying method, logarithm of the ring width (Fig. 4f), and interaction between parameters, were not significant. For K, the random effect variance ($\delta_{T}^{2}$: 9.6 %) was relatively low compared to the fixed effect variance. The total effect variance was 54.86 %, with 45.26 % for the fixed effects and 9.6 % for the random effects (see Table A1 in Appendix). The residuals held a large proportion of the total variance: 45.14 %.

A strong, significant, and positive effect of cambial age on Mn concentration was observed (*p* < 0.001). The Mn concentration was higher in the sapwood than in the heartwood (Fig. 4g). The model also showed a significant relationship between the logarithm of the ring width and Mn concentration. The larger rings had higher Mn concentrations than the narrower rings (Fig. 4h). A significant negative effect was observed between the Mn concentration and the logarithm of the ring width (*p* < 0.001). The random variance represented 45.81 % of the total variance. The random variance was primarily significant for the inter-tree effect of relative cambial age ($\delta_{T_{Ar}}^{2}$: 31.9 %). Fixed effects accounted for 29.49 % of the total variance (see Table A1 in Appendix).

### 3.3 Modeling chain for calculating nutrient translocation

**Fig. 5:**
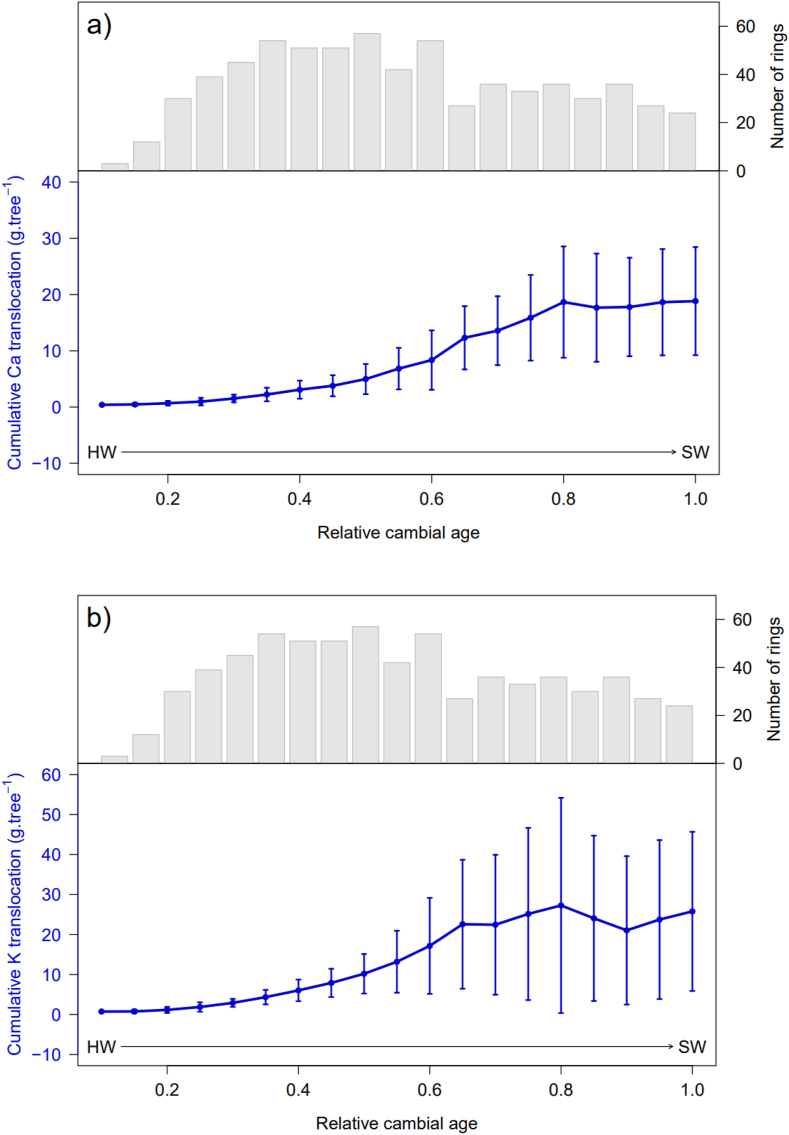

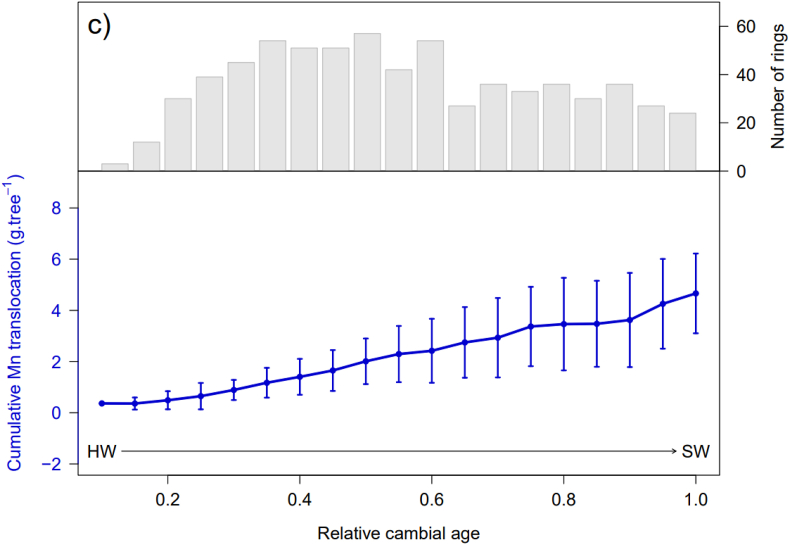
Cumulative translocation of Ca, K, and Mn from the first ring counted on the sample to the age of the trees when the cores were taken. The arrow indicates the direction of radial position of the tree, from heartwood to sapwood. The curve represents the average over nine trees of cumulative translocation for all drying methods combined, knowing that the average age of the nine trees is 39 years. The translocation value at 1 on the x axis indicates the mean total translocation of the nine trees near the bark.

Fig. 5a, **b** and **c** show the normalized mean cumulative Ca, K, and Mn translocations for all the drying methods combined and the number of rings measured for each relative cambial age. Knowing that the values in g tree^−1^ are calculated values, the total translocation amounted to 18.8 ± 9.6 g tree^−1^ Ca, 25.8 ± 15.9 g tree^−1^ K and 4.6 ± 1.5 g tree^−1^ Mn, on average for the nine trees sampled. For each element, the standard deviation was high, indicating high inter-tree variability in translocation. Trees with a larger diameter would also have a higher tree cumulative translocation. A significant effect of the relative cambial age for Ca, K, and Mn (*p* < 0.001, ***, Fig. 5) was observed for tree cumulative translocation which amounted from 0.4 to 17.6 g tree^−1^ between 0 and 0.8 relative cambial age for Ca, 0.7–22.5 g tree^−1^ between 0 and 0.65 relative cambial age for K and 0.3–4.6 g tree^−1^ between 0 and 1 relative cambial age for Mn. This gradually increasing trend indicates that the older rings close to the pith allocate a substantial amount of nutrients to the newly formed rings. For Ca and K, the tree ring translocation of some individuals decreased after 0.8 relative age. This shows that there was an accumulation of these elements in the rings from this relative cambial age. The cumulative translocation per ha was 10.8 ± 5.5 kg ha^−1^ Ca, 14.8 ± 11.4 kg ha^−1^ K and 2.6 ± 0.9 kg ha^−1^ Mn.

## 4 Discussion

### 4.1 Nutrient concentration in oak

The Ca, K, and Mn contents reported in this study were approximately of the same order of magnitude as those reported in other studies. Mohammadzadeh et al. [52] found substantially lower K and Ca contents in *Quercus brantii* sapwood than in the present study and other studies (Table 2). Nutrient content in trees is often strongly related to the pool of available elements in the soil [53,54]. Therefore, low element concentrations in the tree rings may reflect low pools of available soil nutrients, which may be the case in the study of Mohammadzadeh et al. [52].

**Table 2:** Comparison of Ca, K and Mn content in heartwood and sapwood (g kg^−1^) between previous published studies and the present study. The superscript numbers represent the drying method for the samples: (1) drying at ≤ 65 °C (2) drying at 103 °C, and (3) freeze-dried. The (*) next to the reference indicates that the concentrations values are estimated from a graph.

	Mean nutrient concentration (g kg^−1^)			Stand age (years)	Species	Site	References
	Ca	K	Mn				
**Heartwood**	0.38^(1)^	0.65^(1)^		60	*Quercus robur*		[54]*
	0.2^(1)^	0.5^(1)^	0.015^(1)^	130–160	*Quercus robur*		[29]*
	0.28^(2)^	0.5^(2)^	0.07^(2)^	64–129	*Quercus petraea*		[28]
	0.57	0.67	–	100	*Quercus robur*		[55]
	0.62^(1)^	0.9^(1)^	–	–	*Quercus petraea*	B	[58]
	0.45^(1)^	1.06^(1)^	–			W	
	0.62^(3)^	0.602^(3)^	0.036^(3)^	37	*Quercus* spp.		[30]
	0.51^(1)^	0.49^(1)^	0.02^(1)^	100	*Quercus* spp.		[57]
	0.3^(1)^	0.37^(1)^	0.02^(1)^	40	*Quercus* spp.		Present study
	0.29^(2)^	0.37^(2)^	0.02^(2)^				
	0.34^(3)^	0.41^(3)^	0.03^(3)^				
**Sapwood**	0.53^(1)^	1.7^(1)^	–	60	*Quercus robur*		[54]*
	0.55^(1)^	1.25^(1)^	0.13^(1)^	130–160	*Quercus robur*		[29]*
	0.63^(1)^	1.57^(1)^	0.271^(1)^	80–140	*Quercus robur*		[59]
	0.54^(1)^	1.2^(1)^	0.19^(1)^	64–129	*Quercus petraea*		[28]
	0.5	1.65	–	100	*Quercus robur*		[55]
	1.4^(1)^	1.8^(1)^	–	–	*Quercus petraea*	B	[58]
	1.04^(1)^	2.07^(1)^	–			W	
	0.16^(1)^	0.17^(1)^	–		*Quercus brantii*		[52]
	0.86^(3)^	1.14^(3)^	0.05^(3)^	37	*Quercus* spp.		[30]
	0.76^(1)^	1.44^(1)^	0.04^(1)^	100	*Quercus* spp.		[57]
	0.52^(1)^	1.04^(1)^	0.06^(1)^	40	*Quercus* spp.		Present study
	0.45^(2)^	1.09^(2)^	0.05^(2)^				
	0.5^(3)^	1.07^(3)^	0.06^(3)^				

According to the results in Table 1 and Fig. 3, both the XRF and ICP-OES analysis showed that the Ca, K, and Mn contents in the sapwood were significantly higher than those in the heartwood. Except for Ca in the case of the *Quercus robur* L. in Krutul et al. [55], this increasing trend was also reported in other studies on oaks [[28], [29], [30], [31],^54^,[56], [57], [58], [59]] (Table 2). This trend is explained by nutrient translocation that occurs during heartwood formation [13,17,[60], [61], [62]].

Given that K is responsible for osmoregulation, which is important for cell extension, stomatal movement, and other metabolic activities, it is predominantly present in the symplasts of parenchyma rays and has high mobility [10,63]. This enables easy remobilization of K from the senescing sapwood toward the cambium [64]. Ca is a secondary messenger that allows plants to regulate developmental processes in response to environmental stimuli and plays a major role in cell wall and membrane stabilization. Marschner [10] suggested that Ca has low mobility in plants because large amounts are trapped in the cell wall and membrane. The results of this study and other studies on oak are not in line with this low mobility pattern. Ca showed an increasing trend in elemental content from the pith to the sapwood. This could be partly explained by the adsorption of Ca on the cationic exchange capacity of wood, which increases outward from the heartwood of the oak tree [65].

Mn is an important micronutrient for redox systems, as an activator of various enzymes, and for lignin synthesis [10]. As for K and Ca, the Mn concentration was also higher in the sapwood. However, the concentration values differed widely depending on the study. The Mn concentrations in the present study were considerably lower than those reported by André et al. [28] and Balboa-Murias et al. [59]. However, they were in line with the same orders of magnitude of Mn concentrations in heartwood and sapwood found in other studies [30,57] (Table 2). It may be hypothesized that the Mn content in tree rings may be related to its availability and mobility in soils. High Mn concentrations in woods/rings are often reported in acidic soil. Here, this nutrient is more mobile, which could promote uptake by trees [66].

### 4.2 Effect of drying method on mean ring density and nutrient concentration

Whether the cores were dried at 103 °C or 65 °C or freeze-dried, the wood density and nutrient concentration measurements did not vary significantly. Nevertheless, the model for Ca indicated an interaction effect between relative cambial age and the freeze-drying method (*p* < 0.001, ***, Table 1). This effect is primarily attributed to the lower concentration of Ca in the freeze-dried cores than in the cores dried at 103 °C for the relative cambial age between 0.4 and 0.7. This implies that for rings with the same cambial age, freeze-dried rings have a lower Ca concentration than oven-dried rings. Freeze-drying is expected to be the most conservative drying method. It may be hypothesized that an accumulation of nutrients in the middle of the core may occur during oven drying than during freeze-drying. However, further studies are needed to confirm this hypothesis. For oak trees, the use of different drying methods for Ca, K, and Mn did not affect wood density and nutrient concentration. The findings of this study have highlighted that a meta-analysis combining the results from studies that have applied different drying methods can be used safely.

### 4.3 Total nutrient translocation

In this study, the results on Ca showed approximately the same Ca translocation per year as Sette et al. [67], and Ranger et al. [68], that is, 0.3 kg ha^−1^ per year in this study versus 0.4 kg ha^−1^ per year in *Eucalyptus grandis* and in *Pseudotsuga menziesii*, respectively. However, the Ca translocation in the present study was five times lower than the results of Turner and Lambert [69] and Dambrine et al. [70] for *Eucalyptus grandis* and *Picea abies* respectively. It was two times lower than the results of Laclau et al. [8] and Colin-Belgrand et al. [13] for *Eucalyptus PF1* and *Castanea sativa*, respectively. For K translocation, it reached 0.38 kg ha^−1^ per year in the present study. This is substantially lower than the values reported by the other studies [8,9,13,[67], [68], [69], [70]]. Several explanations can be formulated for the differences observed between our results and those of previous studies. The translocation values in this study may be considered at the stand level. The sampled trees were suppressed (see Supplementary Material), likely leading to an underestimation of translocation. This may also partly explain the differences observed in other studies. The translocation of K and Ca in this study was almost of the same order of magnitude. Meanwhile, in other studies [8,9,13,[67], [68], [69], [70]] (Table 3), the amount of K translocated per year was considerably higher than that of Ca. This could be because of the autecology of oak, which may translocate a high quantity of Ca compared to other species, because no data were found in the literature for oak translocation. This hypothesis must be confirmed by further studies. These discrepancies may be attributed to soil fertility levels, which may affect the translocation rate [71,72].

**Table 3:** Total nutrient translocation (kg ha^−1^) in various species.

	Species	Ca	K	Mn	Stand age (years)	References
Annual translocation from the stemwood (kg ha^−1^ per year)	*Eucalyptus grandis*	1.5	6.8	_	27	[69]
	*Pinus sylvestris*	_	6.5	_	46	[9]
	*Picea abies*	1.4	2.9	_	85	[70]
	*Castanea sativa*	0.7	5.8	_	20	[13]
	*Pseudotsuga menziesii*	0.4	1.1	_	60	[68]
	*Eucalyptus PF1*	0.8	11.8	_	7	[8]
	*Eucalyptus grandis*	0.4	4.3		4	[67]
	*Quercus* spp.	0.3	0.38	0.05	40	Present study

Concerning Mn, the translocation in this work reached 0.05 kg ha^−1^ per year, which is substantially lower than translocation of K and Ca, that is, macronutrients, and may be explained by Mn being a micronutrient. However, this result for Mn cannot be compared with those of other studies because none of them report data on Mn.

To fit the nutrient dynamics model, there was a problem with unknown values for the parameters *k* and *b* used in Equation (5) and Equation (6). This arose because diachronic data, that is, several measurements of the same soil at different ages, are missing. As explained in Section 2.6.2, these values were fixed arbitrarily based on expertise to present the full calculation chain for nutrient tree ring translocation for the first time and to check if, with these assumptions on oak parameters, the total calculated translocations were consistent. Table 3 shows that studies dealing with total ring translocation and tree age are relatively scarce, and that this study is the only one that considers oak trees.

A similar trend of cumulative translocation was observed for K and Ca (Fig. 5a, b, c). It was low at the beginning, increased gradually, and flattened when the relative cambial age was approximately 0.65. In contrast, the Mn cumulative translocation gradually increased to the last ring. These trends are primarily linked to (i) increasing tree ring biomass and (ii) tree physiology. Old rings near the pith have a lower biomass than young rings near the bark as the surface area of the tree increases over the years.

### 4.4 Progress made and limit

The ITRAX and ICP-OES devices were both used in this work and enabled obtaining the nutrient content for individual rings. The ICP-OES device provided the total nutrient concentration but this not always at the ring scale. The ITRAX can provide annual resolution data that permit the measurement of nutrient translocation. Therefore, these destructive and nondestructive methods are complementary and useful.

As shown in Fig. 3, the drying method did not affect nutrient translocation values. Nutrient translocations were similar and did not show significant differences among the three drying methods for Ca, K, and Mn. Although this work was based on non-volatile elements, the results may have been different if more volatile elements, such as N, P, and S, had been studied.

## 5 Conclusion

The main objectives of this study were to investigate three methodological issues related to wood translocation.

First, the drying methods tested in this study (65 °C, 103 °C, and freeze-drying) did not affect significantly the concentrations of Ca, K, and Mn in wood cores. Even if these elements are not the most sensitive to drying temperatures, volatile nutrients such as N showed similar results. This suggests that similar measurements can be performed on all wood cores sampled in previous studies and stored after air-drying. Moreover, measuring the translocation of P, Mg, and S would be of interest in determining the extent of translocation of these major elements.

The calibration between nutrient concentrations acquired by chemical analysis by ICP-OES and semi-quantitative values from ITRAX analysis provided Ca, K, and Mn individual tree ring concentrations along the cores. This enabled to obtain nutrient concentrations at the ring scale without grinding. This saves considerable time and enhances the spatial resolution along the tree cores. These results can provide a key reference for future analysis of Ca, K, and Mn translocations in different species from wider geographic areas.

The calculation chain proposed in this study is suitable for any wood core sample and nutrients from species other than oak. On average, the cumulative translocation per hectare of our case study was 10.8 kg ha^−1^ for Ca, 14.8 kg ha^−1^ for K, and 2.6 kg ha^−1^ for Mn and translocation patterns differed according to the elements. Given the cumulative translocation values estimated in this study, the cumulative translocation of nutrients in tree stems may be substantially lower than that in leaves, but further studies have to be conducted to better understand the role of wood translocation in biogeochemical cycles.

## CRediT authorship contribution statement

**Caroline Christina Jaozandry:** Writing – review & editing, Writing – original draft, Visualization, Software, Methodology, Investigation, Formal analysis, Data curation. **Jean-Michel Leban:** Writing – review & editing, Validation, Supervision, Funding acquisition. **Arnaud Legout:** Writing – review & editing, Supervision. **Gregory van der Heijden:** Writing – review & editing. **Philippe Santenoise:** Formal analysis. **Gilles Nourrisson:** Methodology. **Laurent Saint-André:** Writing – review & editing, Validation, Supervision, Resources, Project administration, Methodology, Investigation, Funding acquisition, Conceptualization.

## Declaration of competing interest

The authors declare that they have no known competing financial interests or personal relationships that could have appeared to influence the work reported in this paper.

## Figures and Tables

**Fig. A2:**
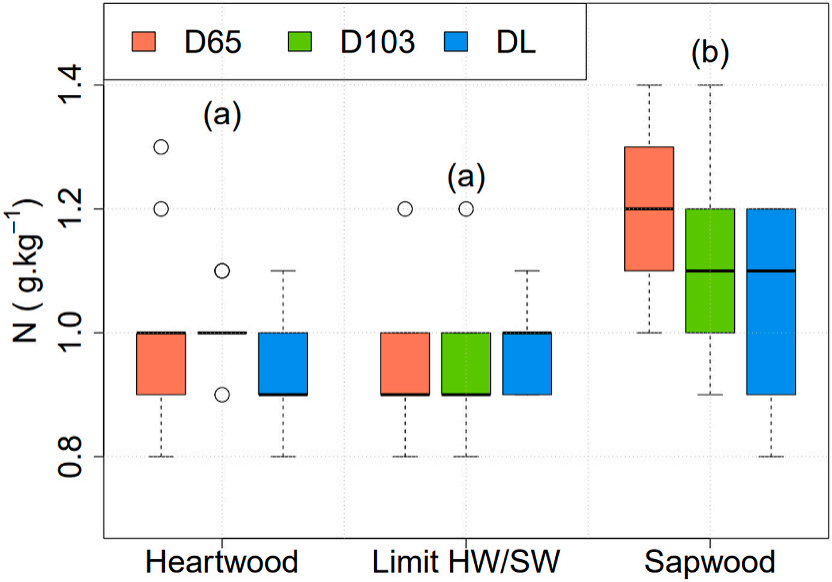
Concentration in N for the three drying methods (D65 = drying at 65 °C, D103 = drying at 103 °C and DL = lyophilization or freeze-dried) according to the radial position in the tree: heartwood, limit HW/SW (transition zone) and sapwood. Different letters indicate a significant difference (p < 0.05; Tukey's Test).

**Table A1:** Contribution of fixed effects and random effects to total variation in mean density and nutrient concentration expressed in % of the total variance $\delta^{2}$. T= Tree, ln (RW) = ln (ring width), Ar= relative cambial age, D65= oven drying at 65 °C and DL = Lyophilization.

		Mean density	Ca	K	Mn
Fixed effects		31.12	37.24	45.26	29.49
Random effects	$\delta_{T}^{2}$	17.52	11.16	9.6	6.5
	$\delta_{T_{\ln\left(RW\right)}}^{2}$	3.65			2.21
	$\delta_{T_{Ar}}^{2}$		19.63		31.9
	$\delta_{T_{D65}}^{2}$	12.72	1.65		3.2
	$\delta_{T_{DL}}^{2}$	14.5	3.48		2
Sum of random effects		48.39	35.92	9.6	45.81
Sum of random and fixed effects		79.51	73.16	54.86	75.3
Residual variance		20.49	26.84	45.14	24.7

## Footnotes

Appendix B Supplementary data to this article can be found online at https://doi.org/10.1016/j.heliyon.2024.e32627.
