# Supplementary material for: Advances in assessing Ca, K, and Mn translocation in oak tree stems (Quercus spp.)
Source: Heliyon. 2024 Jun 13;10(13):e32627. doi: 10.1016/j.heliyon.2024.e32627 (PMC11261777; doi:10.1016/j.heliyon.2024.e32627)
Supplement: Multimedia component 1 [file mmc1.docx]

**Supplementary materials**

1. **Consistency between wood density and nutrient measurement**

For mineral analysis by ITRAX, the relative concentration of each core were measured from the beginning to the end of each core. Thus, this required a conversion matrix core by core to mark the limit of each ring from cross-dated ring width and wood density profiles. Finally, for each core, the wood density profile could be superposed with an index of mineral element concentration ring-to-ring profile. This allowed afterwards the calculation of the amount of nutrient translocated within the tree stem.

1. **Sampled trees caracteristics**

The sampled trees were either suppressed and/or co-dominant one (max. 20 cm DBH) to avoid dilution effects in nutrient concentrations for fast growing trees (dominant and co-dominant (Saint-André et al., 2002)) to keep focused on the methodological issues of this study, and to preserve the integrity of the experimental site which is planned to span more than 30 years.


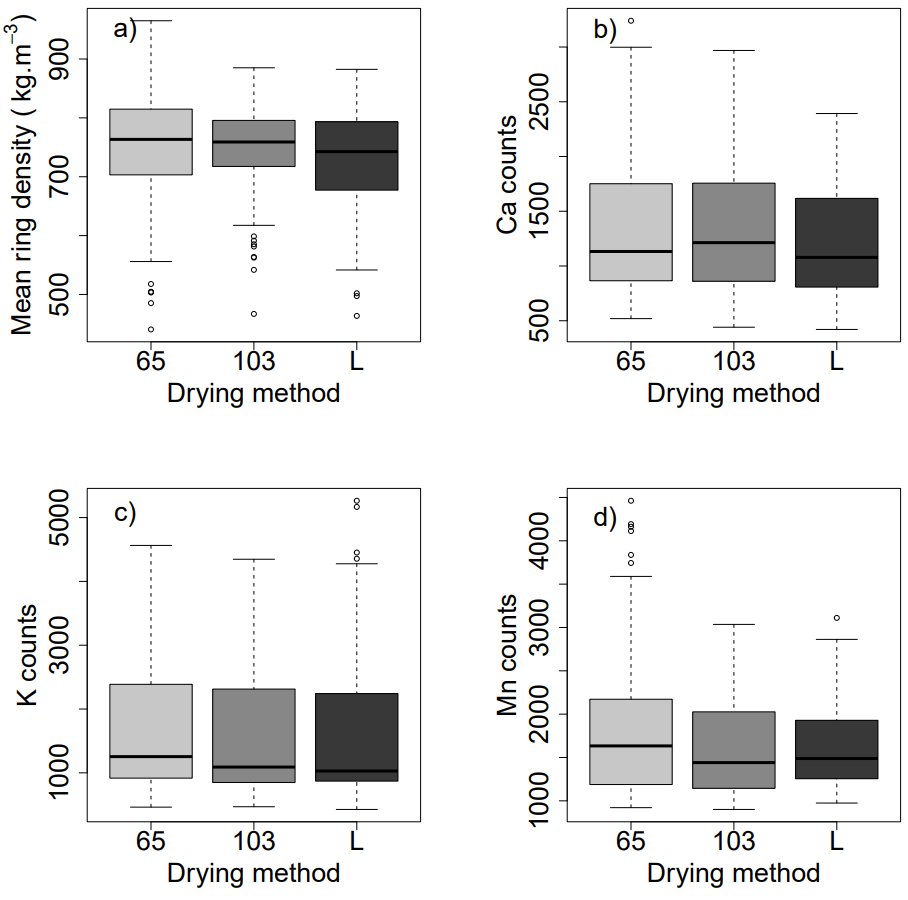


**Fig S. 1.** Ring mean density and nutrients according to the 3 drying methods: a) Ring mean density (kg.m^-3^), b) Calcium counts, c) Potassium counts, d) Manganese counts.


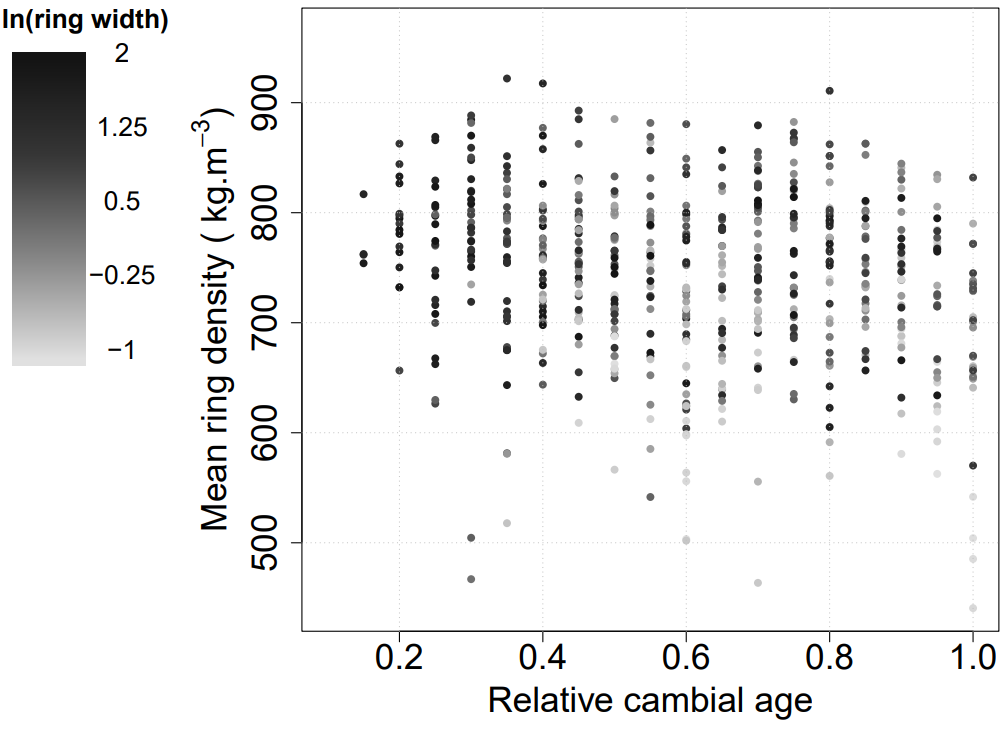

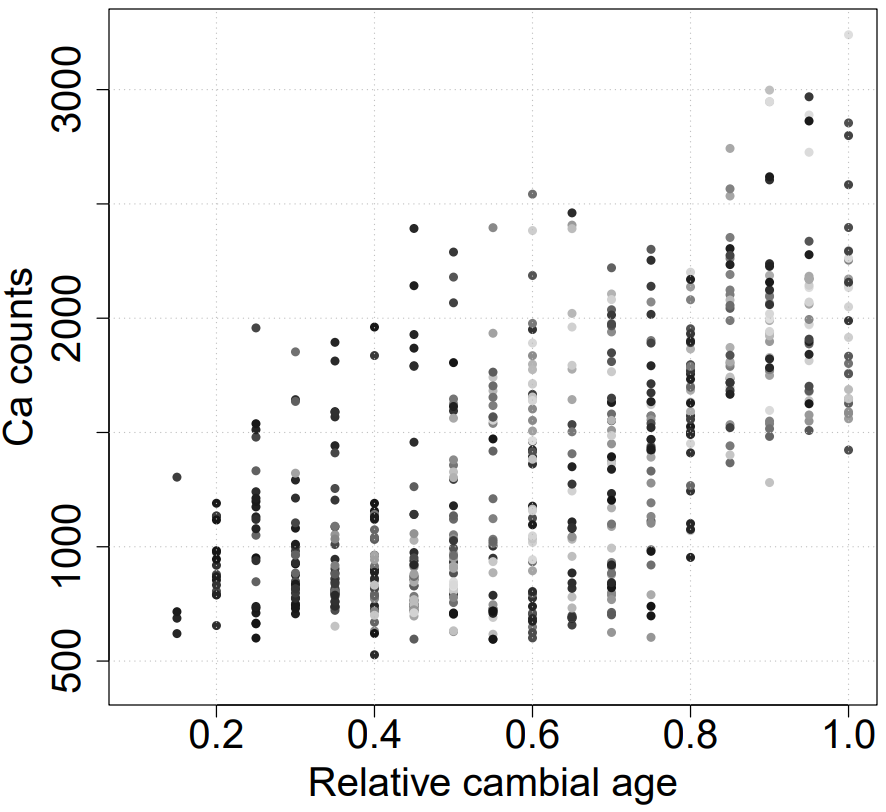


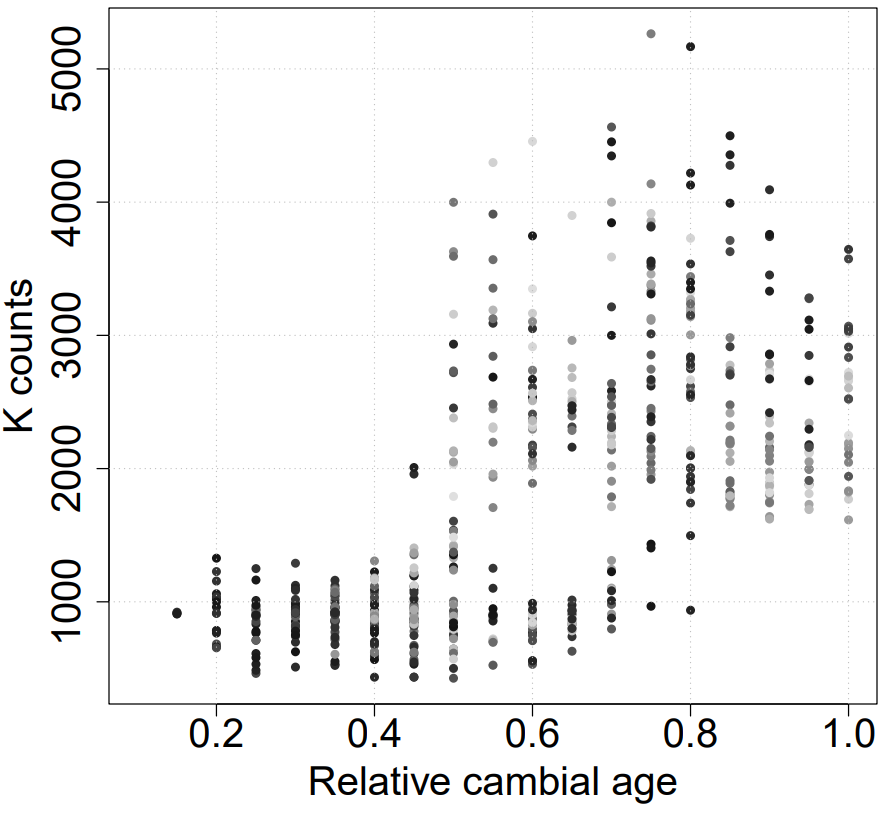

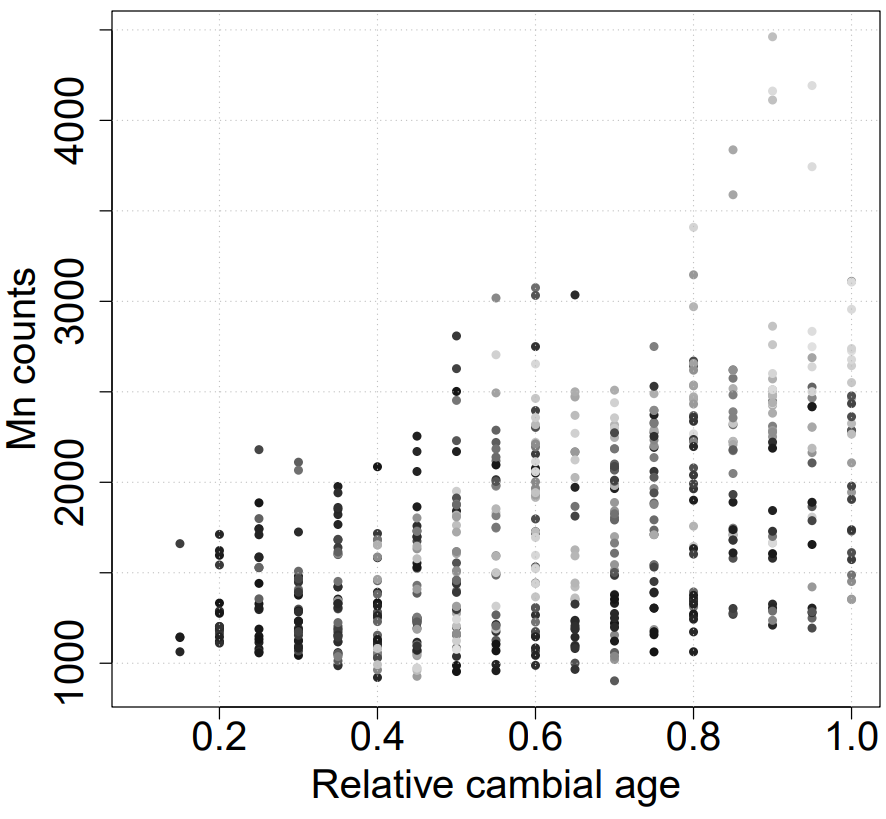


**Fig S. 2.** Ring mean density (kg m^-3^), Ca, K and Mn counts depending on the interaction of relative cambial age and ln(ring width).

**Table S. 1**

Root mean square errors (RMSE), sums of squares errors (SSE), Pearson correlation coefficient (r^2^) and F-tests of the K, Ca and Mn models.

|  |  | **Local K model** | | | **Global K model** |
| --- | --- | --- | --- | --- | --- |
|  |  | **Drying at 103°C** | **Drying at 65°C** | **Freeze-dried** |  |
| **K** | RMSE | 1.84 | 1.84 | 1.89 | 2.92 |
|  | SSE | 3.49 | 3.38 | 3.57 | 8.52 |
|  | p | 1 | 4 | 1 | 2 |
|  | F_obs_ |  |  |  | **0.77** |
|  | F_tab_ |  |  |  | **1.45** |
|  | r^2^ |  |  |  | **0.8** |
| **Ca** | RMSE | 0.47 | 0.54 | 0.49 | 0.85 |
|  | SSE | 0.22 | 0.29 | 0.24 | 0.78 |
|  | p | 2 | 2 | 2 | 2 |
|  | F_obs_ |  |  |  | **0.88** |
|  | F_tab_ |  |  |  | **1.45** |
|  | r^2^ |  |  |  | **0.7** |
| **Mn** | RMSE | 0.1 | 0.11 | 0.12 | 0.19 |
|  | SSE | 0.01 | 0.012 | 0.014 | 0.03 |
|  | p | 4 | 2 | 2 | 4 |
|  | F_obs_ |  |  |  | **0.87** |
|  | F_tab_ |  |  |  | **1.45** |
|  | r^2^ |  |  |  | **0.8** |
